# Metabolomic profiling implicates mitochondrial and immune dysfunction in disease syndromes of the critically endangered black rhinoceros (*Diceros bicornis*)

**DOI:** 10.1038/s41598-023-41508-4

**Published:** 2023-09-19

**Authors:** Molly L. Corder, Emanuel F. Petricoin, Yue Li, Timothy P. Cleland, Alexandra L. DeCandia, A. Alonso Aguirre, Budhan S. Pukazhenthi

**Affiliations:** 1 Smithsonian’s National Zoo and Conservation Biology Institute, Center for Species Survival, Front Royal, 22630 USA; 2 Center for Applied Proteomics and Molecular Medicine, George Mason University, Manassas, 20900 USA; 3 Department of Environmental Sciences and Policy, George Mason University, Fairfax, Virginia 22030 USA; 4 Department of Chemistry and Biochemistry, University of Maryland, College Park, MD 20742 USA; 5 Smithsonian Museum Conservation Institute, Suitland, MD 20746 USA; 6 Department of Biology, Georgetown University, Washington, DC, 20057 USA; 7 Smithsonian’s National Zoo and Conservation Biology Institute, Center for Conservation Genomics, Washington, DC, 20008 USA; 8 Department of Fish, Wildlife, and Conservation Biology, Warner College of Natural Resources, Colorado State University, Fort Collins, 80523 USA

**Keywords:** Animal physiology, Metabolic disorders

## Abstract

The critically endangered black rhinoceros (*Diceros bicornis*; black rhino) experiences extinction threats from poaching in-situ. The ex-situ population, which serves as a genetic reservoir against impending extinction threats, experiences its own threats to survival related to several disease syndromes not typically observed among their wild counterparts. We performed an untargeted metabolomic analysis of serum from 30 ex-situ housed black rhinos (Eastern black rhino, EBR, n = 14 animals; Southern black rhino, SBR, n = 16 animals) and analyzed differences in metabolite profiles between subspecies, sex, and health status (healthy n = 13 vs. diseased n = 14). Of the 636 metabolites detected, several were differentially (fold change > 1.5; *p* < 0.05) expressed between EBR vs. SBR (40 metabolites), female vs. male (36 metabolites), and healthy vs. diseased (22 metabolites). Results suggest dysregulation of propanoate, amino acid metabolism, and bile acid biosynthesis in the subspecies and sex comparisons. Assessment of healthy versus diseased rhinos indicates involvement of arachidonic acid metabolism, bile acid biosynthesis, and the pentose phosphate pathway in animals exhibiting inflammatory disease syndromes. This study represents the first systematic characterization of the circulating serum metabolome in the black rhinoceros. Findings further implicate mitochondrial and immune dysfunction as key contributors for the diverse disease syndromes reported in ex-situ managed black rhinos.

## Introduction

The critically endangered black rhinoceros (*Diceros bicornis*) experiences extinction threats in-situ due to poaching pressures^1^. The extinction risk for wild black rhinos necessitates that self-sustaining ex-situ (zoo or breeding center housed) populations be maintained to serve as a genetic reservoir against impending extinction threats. However, the ex-situ population experiences its own threats to survival related to several unusual disease syndromes not typically observed among their wild counterparts^2,3^. With < 5627 wild black rhinos^4^ and < 240 black rhinos in ex-situ management worldwide^5,6^, the health and welfare of the ex-situ population is a high priority for the conservation community. An understanding of the fundamental physiological mechanisms that contribute to taxonomic divergence, reproductive biology, and disease states is a high priority for maintaining self-sustaining populations of rare and endangered species.

As “canaries of the genome”, metabolites play a key role in modulating the physiology of living beings via their diverse actions as signaling molecules, immune modulators, endogenous toxins, and environmental sensors^7,8^. While endogenous metabolites are largely conserved among populations or species, individual metabolomes can vary based on internal or external variables such as host genome composition, age, sex, diet, geographical locations, environment, and time of day^7^. In conservation medicine, metabolomic profiling could be widely applied to answer physiological questions pertaining to taxonomic divergence, reproductive biology, and health of species threatened with extinction.

The International Union for the Conservation of Nature (IUCN) acknowledges the taxonomic divergence of three extant subspecies of black rhinoceros (*Diceros bicornis):* eastern (*D.b. michalei*), south-eastern (*D.b. minor*), and south-western (*D.b. bicornis*); and one extinct subspecies as of 2011, the western black rhinoceros (*D.b. longipes*)^1^. Recent research validated differences in these distinct evolutionary lineages with genetic variation from mitochondrial and nuclear DNA^9,10^. Metabolomic differences in other taxa have been reported at the species level between naked mole rats and mice^11^, and at the breed level in pigs^12^, cattle^13^, chickens^14^, and dogs^15^. However, there is limited information on serum metabolome of rhinos^16^.

Metabolomics research has greatly enhanced our understanding of the fundamental mechanisms involved in reproduction. In humans, distinct patterns associated with sex have been identified in diverse biofluids^17^. Metabolomic profiling has also been used to predict differences in disease risk based on sex^18^. In humans and model species, non-invasive monitoring of the metabolic activity of embryos in complex culture environments resulted in new knowledge on the species-specific and environmentally significant (*in-vitro* compared to *in-vivo*) requirements of competent embryos^19^. Metabolomic profiling of embryo culture media from *in-vitro fertilization* (IVF) has successfully predicted bovine embryo sex^20,21^. Yet, these research approaches remain largely underutilized for endangered species. Furthermore, generating baseline metabolomic data on the differences between sexes could enhance our understanding of species-specific reproductive mechanisms.

A major challenge to black rhinos in managed care is the high incidence of unusual disease syndromes not typically observed among their wild counterparts^2,3^. These disease syndromes present enormous variation in symptoms and often occur concurrently with more than one disease. An earlier epidemiological survey of the ex-situ black rhino population reported that disease syndromes including hemolytic anemia, leukoencephalomalacia, superficial necrolytic dermatitis, idiopathic hemorrhagic vasculopathy syndrome, and toxic hepatopathy were often observed in zoo-managed rhinoceros^2^. Interestingly, these disease syndromes could not be clearly classified as separate syndromes with distinct etiologies and could reflect the same underlying syndrome with varying manifestations.

Metabolomic profiling is now accepted as the new frontier in clinical chemistry^7^. While routine clinical (serum) chemistry profiles assess a finite number of serum components to evaluate organ function, metabolomic profiling can measure hundreds or thousands of metabolites. The ability to detect and quantify diverse metabolites present in a single sample also advances biomarker discoveries in medicine^22–25^. Metabolomic profiling has been extensively applied in health research. When coupled with other -omics technologies, metabolomics can be a powerful tool for identifying candidate biomarkers that may have physiological significance and may be linked to disease phenotypes^26,27^. Metabolomics has been applied as a tool for characterizing cellular immunometabolism and (subsequently) the discovery of biomarkers involved in immune-mediated disease^28^. In rats, age-related changes have been detected in the gut microbiome and linked (via function analysis) with the serum metabolome and (via correlation analysis) with a network of immune factors – thus describing a clear relationship between immune factors, the serum metabolome, and gut microbiome^29^**.** Fecal metabolomics has detected differences in metabolites between colorectal cancer patients and healthy controls, which resulted in the identification of candidate biomarkers^30,31^. Metabolomics is rarely utilized in veterinary and conservation medicine^32^. However, the fecal metabolome of all four species of rhinoceros has been evaluated^33^. Results indicated that the black rhinoceros expressed a high abundance of short chain fatty acids (2-hydroxybutyrate, butyrate, isobutyrate, isovalerate, and propionate) when compared to the other three species of rhinoceros^33^.

In the present study, we employed an untargeted metabolomics approach to analyze Eastern (EBR) and Southern (SBR) black rhinoceros serum to generate new knowledge that could help explain the physiological basis of the numerous disease syndromes often observed in ex-situ managed black rhinoceros. Specifically, the objectives of this study were to (1) characterize the serum metabolome for the first time in the black rhinoceros, (2) explore differential expression of metabolites based on covariates (subspecies, sex, and health status), and (3) identify potential candidate metabolic pathways contributing to observed disease syndromes for future study. We hypothesized that black rhinos would display differences in the metabolome based on subspecies, sex, and health status. We further hypothesized that differences in metabolite expression between diseased and healthy animals would provide preliminary insight into the molecular networks that may be perturbed in various disease states often reported in ex-situ managed black rhinos.

## Results

### Animals used in this analysis and health histories

A total of 30 black rhinos from 15 US zoological institutions and private ranches were enrolled in this study. The study population comprised of (1) 14 EBR and 16 SBR, (2) 15 females and 15 males, and (3) 13 clinically healthy, 14 inflammatory phenotype, and 3 other/non-inflammatory phenotype. Individuals classified in the “other” phenotype had clinical histories of reproductive dysfunction but no obvious signs of inflammation. Therefore, these animals were omitted from the study. The health survey results revealed that chronic inflammation, ulcerative dermatitis, metabolic disease, hepatic disease, reproductive dysfunction, lameness, dental/periodontal disease, chronic intermittent nose/tail bleeding, and chronic intermittent diarrhea were present in the population (Appendix S1). Animals with a history of chronic inflammation, ulcerative dermatitis, metabolic disease, hepatic disease, lameness, and dental/periodontal disease were grouped into the inflammatory phenotype group. Animals included in the healthy phenotype group (six EBR (1 male and 5 females) and seven SBR (3 males and 4 females) had no reported clinical issues. Only healthy animals were included in the subspecies and sex comparisons.

### Untargeted serum metabolomic profiling

**Figure 1:**
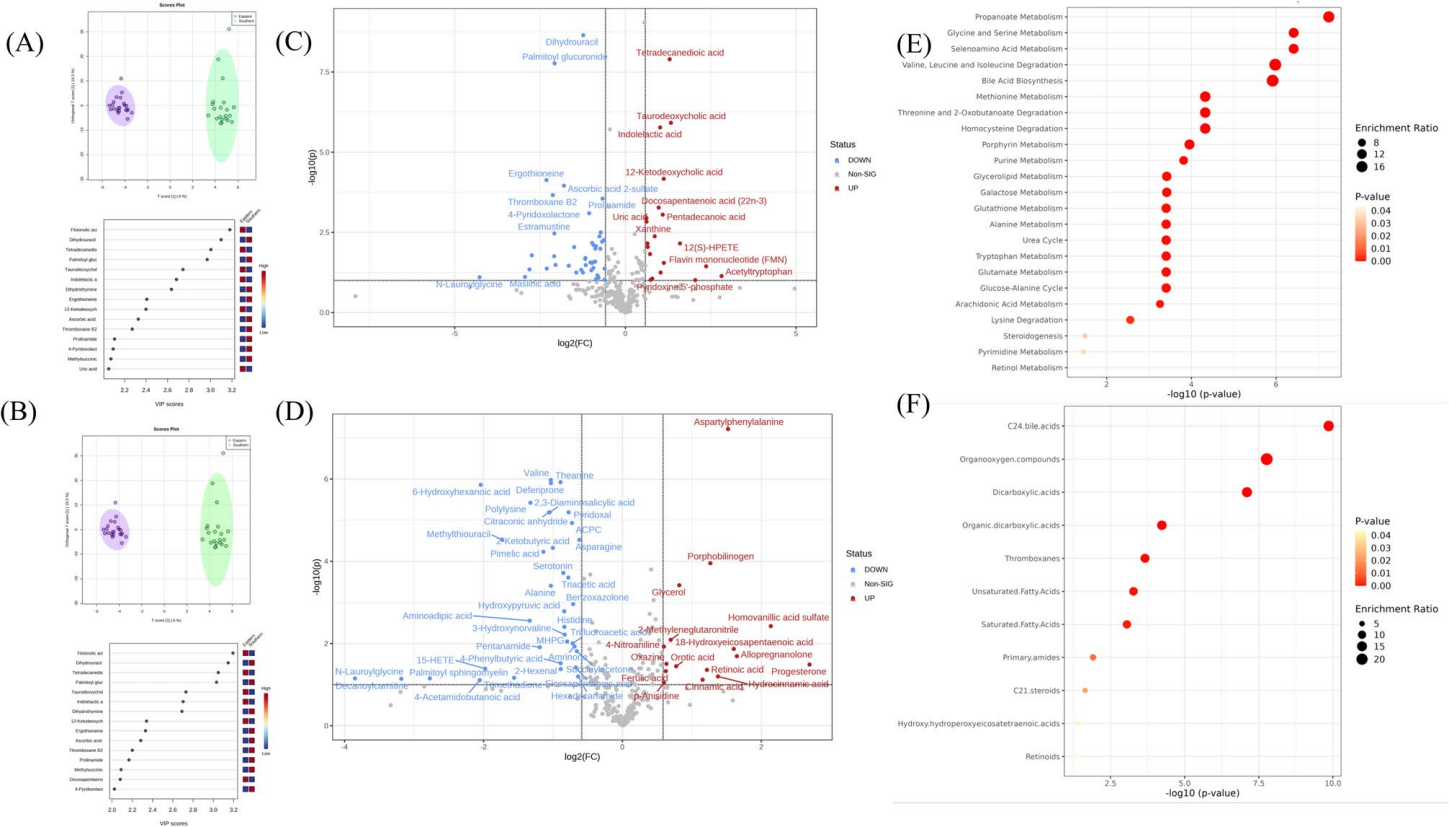
Serum metabolome profile for eastern versus southern subspecies comparison relative to the eastern subspecies. (**A**) ESI− OPLSDA scores plot and VIP scores plot, (**B**) ESI+ OPLSDA scores plot and VIP scores plot, (**C**) ESI− volcano plot of differentially expressed metabolites, (**D**) ESI+ volcano plot of differentially expressed metabolites, (**E**) enriched metabolite pathways, and (**F**) enriched lipid pathways. *Thirteen clinically healthy animals were included in this comparison (six EBR (5.1) and seven SBR (4.3)).

**Figure 2:**
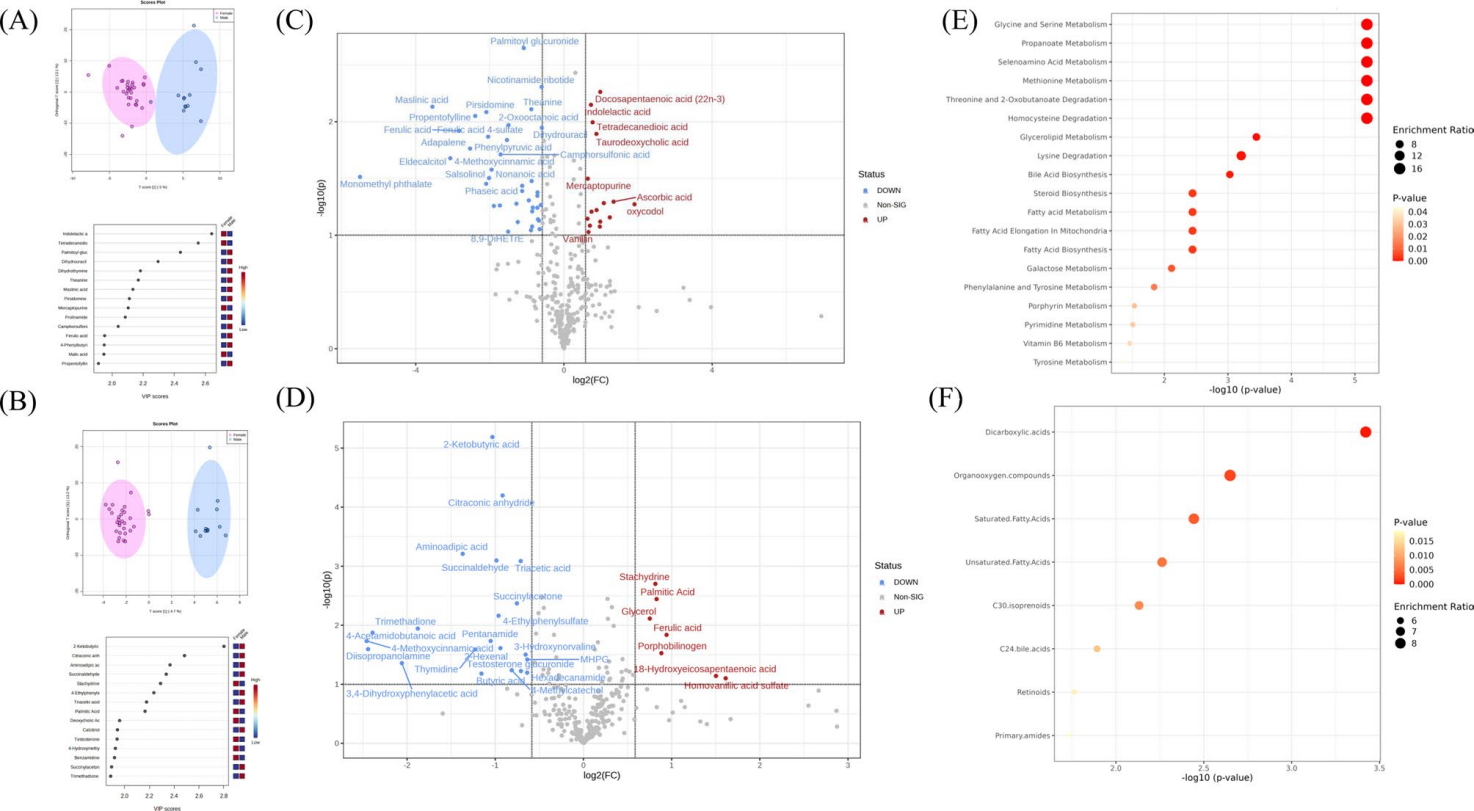
Serum metabolome profile for sex comparison relative to females. (**A**) ESI− OPLSDA scores plot and VIP scores plot, (**B**) ESI+ OPLSDA scores plot and VIP scores plot, (**C**) ESI− volcano plot of differentially expressed metabolites, (**D**) ESI+ volcano plot of differentially expressed metabolites and (**E**) enriched metabolite pathways and (**F**) enriched lipid pathways. *Thirteen clinically healthy animals were included in this comparison (six EBR (1.5) and seven SBR (3.4)).

**Figure 3:**
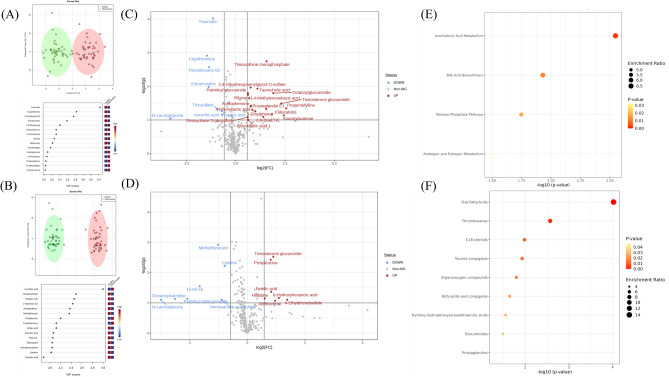
Serum metabolome profile for health phenotype comparison (healthy vs. inflammatory) relative to inflammatory phenotype. (**A**) ESI− OPLSDA scores plot and VIP scores plot, (**B**) ESI+ OPLSDA scores plot and VIP scores plot, (**C**) ESI− volcano plot of differentially expressed metabolites, (**D**) ESI+ volcano plot of differentially expressed metabolites and (**E**) enriched metabolite pathways and (**F**) enriched lipid pathways. *Thirteen clinically healthy animals (six EBR (1.5) and seven SBR (3.4)) were compared with fourteen animals with inflammatory phenotypes (six EBR (5.1) and eight SBR (5.3)).

We identified 636 metabolites that were expressed in all samples (352 ESI− and 284 ESI+). We generated metabolome and lipid profiles (Figs. 1, 2 and 3) and tables of differentially expressed metabolites and lipids (Tables 1, 2 and 3). Differentially expressed compounds met two criteria, first had fold change values of < 0.5 for down-regulated and > 1.5 for up-regulated compounds, and second yielded significant p-values (< 0.05) in the t-test comparison. Only compounds that met both these criteria are reported in Tables 1, 2 and 3.

**Table 1:** Differentially expressed metabolites by subspecies (eastern vs. southern). Fold change values are relative to eastern black rhinoceros subspecies.

	t. stat	*p* value	FC
Source ESI−			
Taurodeoxycholic acid•	5.6855	1.21E−06	2.52131958
Tetradecanedioic acid•	7.0819	1.26E−08	2.45810676
12-Ketodeoxycholic acid•	4.4349	6.76E−05	2.18009379
Pentadecanoic acid•	3.5855	0.00088697	2.13582382
Indolelactic acid	5.5838	1.69E−06	2.02595388
Docosapentaenoic acid (22n-3)•	3.7582	0.0005339	1.96740629
Xanthine	3.4112	0.0014654	1.53526241
Uric acid	3.5014	0.0011315	1.53262735
4-Pyridoxolactone	− 3.621	0.00079968	0.47959939
Dihydrouracil	− 7.6175	2.25E−09	0.42519574
Ascorbic acid 2-sulfate	− 4.2768	0.00011058	0.2872719
Palmitoyl glucuronide•	− 6.9899	1.70E−08	0.23807027
Thromboxane B2•	− 4.056	0.00021803	0.22906063
Ergothioneine	− 4.406	7.40E−05	0.20150888
Source ESI+			
Progesterone*•	2.2134	0.032494	6.47124629
Homovanillic acid sulfate	3.0715	0.0037737	4.39691326
Allopregnanolone•	2.4143	0.020312	3.13438286
18-Hydroxyeicosapentaenoic acid	2.5804	0.013547	3.03788485
Aspartylphenylalanine	6.6006	6.06E−08	2.87004594
Porphobilinogen	4.2751	0.00011119	2.40478626
Retinoic acid•	2.08	0.043814	2.32514366
Glycerol	3.8697	0.0003829	1.76333235
Orotic acid	2.172	0.035689	1.71008204
2-Methyleneglutaronitrile	2.7814	0.0081408	1.61873648
Oxazine	2.23	0.031281	1.55195428
Ferulic acid	2.0482	0.046976	1.54191347
4-Nitroaniline	2.6325	0.011895	1.51281985
2-Ketobutyric acid	− 4.5491	4.72E−05	0.49954186
Deferiprone	− 5.6743	1.26E−06	0.48999963
Alanine	− 3.8595	0.00039486	0.4894637
Valine	− 5.7294	1.05E−06	0.4890865
2,3-Diaminosalicylic acid	− 5.1713	6.45E−06	0.48388992
Citraconic anhydride	− 5.1704	6.47E−06	0.48066615
Pimelic acid•	− 4.4788	5.89E−05	0.45459926
Pentanamide•	− 2.6177	0.012345	0.43866384
Polylysine	− 5.3375	3.77E−06	0.39933318
Aminoadipic acid•	− 3.1819	0.0027889	0.39649309
Methylthiouracil	− 4.6962	2.96E−05	0.30091508
15-HETE•	− 2.1102	0.040991	0.254377
6-Hydroxyhexanoic acid	− 5.6445	1.38E−06	0.24319717

•Indicates lipids.

*Progesterone is likely not differentially expressed by subspecies. Thirteen healthy animals were included in this comparison: seven SBR and six EBR. Five out of six EBR were females, which presumably drove the differential expression of progesterone in this comparison.

**Table 2:** Differentially expressed metabolites by sex (female vs. male). Fold change values are relative to females.

	t. stat	*p* value	FC
Source ESI−			
Docosapentaenoic acid (22n-3)•	2.9338	0.0054609	1.97305907
Taurodeoxycholic acid•	2.6031	0.012804	1.83623317
Tetradecanedioic acid•	2.6967	0.010114	1.71180669
Indolelactic acid	2.8343	0.0070971	1.65798475
Mercaptopurine	2.2233	0.031769	1.5620004
Palmitoyl glucuronide•	− 3.2607	0.0022406	0.47181734
Phaseic acid	− 2.1599	0.036678	0.45798231
5-Thymidylic acid	− 2.1124	0.040787	0.45775394
2-Oxooctanoic acid	− 2.674	0.010713	0.35461647
Phenylpyruvic acid	− 2.553	0.014498	0.34461674
Camphorsulfonic acid	− 2.4331	0.019417	0.30588535
4-Methoxycinnamic acid	− 2.3028	0.026438	0.25840758
Salsolinol	− 2.2291	0.031351	0.24644438
Ferulic acid 4-sulfate	− 2.5798	0.013567	0.24199973
Pirsidomine	− 2.7774	0.0082248	0.2343374
Pyridoxine 5'-phosphate	− 2.1775	0.035252	0.23348632
Propentofylline	− 2.7471	0.0088926	0.1901483
Adapalene•	− 2.4824	0.017237	0.17275389
Ferulic acid	− 2.628	0.012029	0.1404379
Eldecalcitol	− 2.4008	0.020978	0.1192876
Maslinic acid•	− 2.8195	0.0073755	0.08544281
Monomethyl phthalate	− 2.2391	0.030636	0.02205201
Source ESI+			
Ferulic acid	2.5517	0.014544	1.92205649
Porphobilinogen	2.2525	0.02971	1.84510842
Palmitic acid•	3.0886	0.0036017	1.77700029
Stachydrine	3.3028	0.0019916	1.76008879
Glycerol	2.8028	0.0077018	1.68436734
2-Ketobutyric acid	− 5.1703	6.47E−06	0.48896395
Pentanamide•	− 2.4536	0.018483	0.48227822
Thymidine	− 2.3162	0.025626	0.42664645
Aminoadipic acid•	− 3.7096	0.0006165	0.38719999
Trimethadione	− 2.6502	0.011378	0.27198445
3,4-Dihydroxyphenylacetic acid	− 2.0791	0.043907	0.2400207
4-Acetamidobutanoic acid	− 2.5842	0.013421	0.19060806
Diisopropanolamine	− 2.3201	0.025389	0.18405226
4-Methoxycinnamic acid	− 2.4533	0.018494	0.18201671

•Indicates lipids.

**Table 3:** Differentially expressed metabolites by health phenotype (inflammatory vs. healthy). Fold change values are relative to the inflammatory health phenotype.

	t. stat	*p* value	FC
Source ESI−			
Propentofylline	− 2.0348	0.045028	5.88792017
Octanoylglucuronide	− 2.4595	0.015963	3.73329362
Thioxanthine monophosphate	− 3.2229	0.0018073	2.95761862
Prostaglandin E1•	− 2.0102	0.047623	2.91518864
Taurocholic acid•	− 2.576	0.011745	2.14350998
3-Ethylphenol	− 1.9937	0.049427	1.78495476
3-Phenyllactic acid	− 2.0891	0.039729	1.70172513
Testosterone glucuronide•	− 2.1765	0.032326	1.69973094
Acetophenone	− 2.1118	0.037671	1.69973094
3,4-Dihydroxyphenylglycol O-sulfate	− 2.6046	0.010877	1.68968524
3,4-Methylenesebacic acid•	− 2.3218	0.022666	1.6017948
Palmitoyl glucuronide•	− 2.4613	0.015891	1.53747944
Ribose	− 2.4159	0.017866	1.53392427
Traumatin•	4.0985	9.55E−05	0.44957377
Thromboxane B2•	3.0929	0.0026906	0.3915026
Estramustine•	2.6219	0.010379	0.38657356
Ergothioneine	3.3455	0.0012296	0.36414648
Source ESI+			
Testosterone glucuronide	− 3.0583	0.0029856	1.86523858
Ferulic acid	− 2.0548	0.043004	1.77684402
Prolylleucine	− 2.9843	0.0037213	1.7504322
Methylthiouracil	3.3544	0.0011951	0.4944294
15-HETE•	2.2363	0.027984	0.31832483

•Indicates lipids.

### Metabolic profiling of SBR vs EBR

Orthogonal Partial Least Squares Discriminant Analysis (OPLS-DA) scores plots showed differences in metabolites between subspecies (Fig. 1A and B). Forty metabolites were differentially expressed between subspecies (Table 1 and Fig. 1C and D). Quantitative analysis of enriched metabolite pathways (positive and negative ion modes combined) revealed the involvement of 22 pathways (Fig. 1E). The most enriched pathways included metabolism of propanoate, glycine and serine, selenoamino acid, degradation of valine, leucine, and isoleucine, and bile acid biosynthesis. Analysis of enriched lipid pathways (positive and negative ion modes combined) revealed the involvement of 11 pathways (Fig. 1F). The most enriched pathways included C24 bile acids, organooxygen compounds, dicarboxylic acids, organic dicarboxylic acids, and thromboxanes.

### Metabolic profiling of female vs. male black rhinos

Score plots from OPLS-DA showed differences in metabolites between sexes (Fig. 2A and B). Thirty-six metabolites were differentially expressed between sexes (Table 2 and Fig. 2C and D). Quantitative analysis of enriched metabolite pathways (positive and negative ion modes combined) revealed the involvement of 19 pathways (Fig. 2E). The most enriched pathways included metabolism of glycine and serine, propanoate, selenoamino acid, methionine, and degradation of threonine and 2-oxobutanoate. Analysis of enriched lipid pathways (positive and negative ion modes combined) revealed the involvement of 8 pathways (Fig. 2F). The most enriched pathways included dicarboxylic acids, organooxygen compounds, saturated fatty acids, unsaturated fatty acids and C30 isoprenoids.

### Metabolic profiling of inflammatory phenotype vs. healthy black rhinos

Score plots from OPLS-DA showed differences in metabolites between inflamed and healthy groups (Fig. 3A and B). Twenty-two metabolites were differentially expressed between inflamed and healthy groups (Table 3 and Fig. 3C and D). Quantitative analysis of enriched metabolite pathways (positive and negative ion modes combined) revealed the involvement of four pathways (Fig. 3E). The most enriched pathways included arachidonic acid metabolism, bile acid biosynthesis, pentose phosphate pathway, and androgen and estrogen metabolism. Analysis of enriched lipid pathways (positive and negative ion modes combined) revealed the involvement of nine pathways (Fig. 3F). The most enriched pathways included oxo fatty acids, thromboxanes, C18 steroids, taurine conjugates, and organooxygen compounds.

### Biomarker analysis

**Figure 4:**
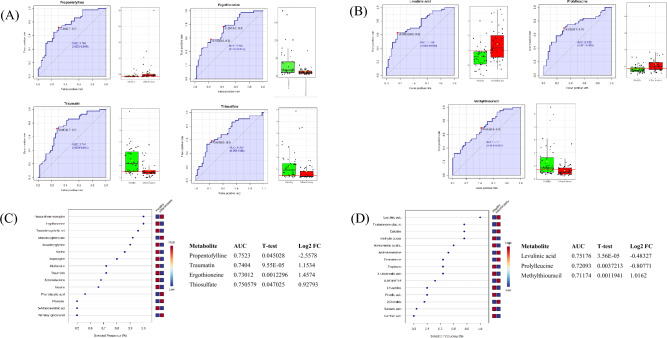
Receiver operating characteristic (ROC) curve-based approach for identifying potential biomarkers and evaluating their performance using area under the curve (AOC). Seven potential/candidate biomarkers were detected: (**A**) four metabolites from the ESI− and (**B**) three from the ESI+ datasets. (**C**) and (**D**) Both datasets were fed into a supervised classification algorithm using the support vector machine classification method with the SVM built-in ranking method to determine which metabolites contributed to significant features using selected frequency %.

Seven potential candidate biomarkers were identified for diagnosis of inflammatory phenotypes using the biomarker analysis feature in the MetaboAnalyst software^34^. This approach relies on a receiver operating characteristic (ROC) curve to identify candidate biomarkers based on their performance using area under the curve (AOC). All seven metabolites also showed significant (*p* < 0.05) features using machine learning via the SVM built-in ranking method. Although larger studies report optimal AUC values > 0.8 for biomarker analysis, we detected AUC values > 0.7 in our relatively small cohort of individuals (Fig. 4). Thus, this biomarker identification algorithm indicates that these seven compounds could potentially serve as biomarkers for differentiating between clinically healthy and inflammatory phenotypes (animals).

## Discussion

Black rhinos managed in human care experience a myriad of poorly understood disease syndromes not reported in their wild counterparts. Routine clinical assessments often fail to reveal the pathophysiological basis of these diseases. This has warranted the application of more sensitive analytical approaches including mass spectrometry (metabolomics). In this study, we analyzed the serum metabolome profile of black rhinos derived from two subspecies (EBR and SBR) and examined differences in expression of metabolites between sexes and healthy vs. diseased (inflammation) animals. This represents the first characterization of the circulating serum metabolome of black rhinoceros. Although significant differences were detected between subspecies and sexes, our current analysis revealed for the first time, perturbations in several metabolic pathways involved in mitochondrial and immune function in diseased rhinos. This new knowledge could facilitate the discovery and validation of novel serum biomarkers for disease diagnosis and treatment.

Within the subspecies comparison, the most enriched pathways included (1) propanoate metabolism, (2) glycine and serine metabolism, (3) selenoamino acid metabolism, and (4) valine, leucine, and isoleucine degradation. Only clinically healthy animals were included in the subspecies comparison. These thirteen animals consisted of six EBRs and seven SBRs. While the differences observed between the subspecies could be attributed to taxonomic divergence, it is more likely the result of different management conditions. The southern subspecies is primarily housed in ranches with consistent access to diverse browse materials in their diet. In contrast, EBRs were housed in traditional zoos distributed among different geographic and climatic zones with more limited access to good quality browse materials. It is plausible that the difference in diet may have contributed to the metabolomic profile differences between subspecies. Additionally, the SBR population was generally “older” than the EBR population (Appendix S5 and S6). Therefore, it is likely that ageing contributes to increased risk for developing disease syndromes and may account for differences observed in metabolomic profiles. Further, differences in husbandry practices can impact stress levels of captive animals and animals with higher stress levels can have an increased risk of developing disease syndromes^35^. This phenomena has been extensively studied in other wildlife species^36–38^. Previous research on black rhinos housed in traditional zoo environments identified that social stressors are potential causes of chronic stress in black rhinos, and may be associated with biological costs that contribute to captive-population sustainability problems^39^. Later research determined that alterations in adrenal activity was associated with inflammatory disease phenotypes in black rhinos^40^. Thus, there are many contributing factors that could be driving differences observed in the subspecies comparison.

Comparison of serum metabolites between male and female black rhinos revealed enrichment of four pathways including (1) glycine and serine metabolism, (2) propanoate metabolism, (3) selenoamino acid metabolism, and 4) methionine metabolism. Of the thirteen animals included in this comparison, nine animals were female (five EBR and four SBR) and four were male (one EBR and three SBR). It is likely that the observed sex-specific differences may be confounded by the skew in our study population (Appendix S5 and S6). Interestingly, the enriched pathways from both the subspecies and sex comparisons resembled the metabolomic profiles of the inflammatory phenotype. This may suggest that several of the animals deemed clinically healthy by clinicians and animal managers may be experiencing subclinical (not overt) signs of disease. Amino acids are involved in modulation of immune function^41^ and dysregulation of amino acid metabolic pathways has been reported in human patients affected by bacterial infections^42^. It is unclear how many rhinos in this comparison were already diseased. These findings highlight the need to develop more reliable and sensitive diagnostic tools (biomarkers) for assessing black rhino health.

Our most robust comparison included fourteen rhinos with histories of inflammatory disease to thirteen clinically healthy rhinos. Animals associated with the inflammatory phenotype exhibited perturbations in three major metabolic pathways including (1) arachidonic acid, (2) bile acid biosynthesis, and the (3) pentose phosphate pathway. We further identified lipid pathways that were perturbed in the inflammatory phenotype including (1) fatty acids and conjugates (oxo fatty acids), (2) eicosanoids (thromboxanes), (3) steroid conjugates (C18 steroids), and (4) fatty aryls (taurine conjugates). Relative to the inflammatory phenotype group, the most substantially up-regulated compounds included: propentofylline, octanoylglucuronide, thioxanthine monophosphate, prostaglandin E1, and taurocholic acid; while the most substantially down-regulated compounds included traumatin, methylthiouracil, ergothioneine, ribose, and thromboxane B2 (Table 3). Further, the MetaboAnalyst^34^ biomarker analysis indicated that propentofylline, traumatin, ergothioneine, thiosulfate, levulinic acid, prolylleucine, and methylthiouracil may serve as potential biomarkers for inflammatory disease syndromes in this species (Fig. 4).

Arachidonic acid is a polyunsaturated fatty acid that can act as an inflammatory intermediate, and has been implicated in immune modulation, cell signaling, and oxidative stress^43^. Further, alterations to the arachidonic acid metabolism pathway have been linked to disease syndromes including inflammatory bowel disease^44,45^, sepsis^46^ and inflammatory shock syndrome^47^, insulin resistance^48^, and mitochondrial dysfunction^49–51^. Recent work suggests that inflammation in inflammatory bowel disease may be a consequence of the disruption of mechanisms that regulate the inflammatory response^44^. In the present study, we detected perturbations in arachidonic acid metabolism in diseased animals via the differential expression of Thromboxane B2 and 15(S)- hydroxyeicosatetraenoic acid (HETE) (see Table 3). Our data provides for the first time in rhinos, evidence of differential expression of pro-inflammatory lipid mediators that are known to be formed from arachidonic acid including HETE, prostaglandins, and thromboxanes. Further, in nonalcoholic fatty liver disease mouse models thromboxane A2 (precursor to thromboxane B2) has been shown to contribute to insulin resistance by impairing insulin signaling^52^. Previous studies in black rhinos have suggested the possible dysregulation of insulin signaling in diseased animals^53^ and warrants additional research to examine the molecular mechanisms perturbed in diseased black rhinos^52^.

**Figure 5:**
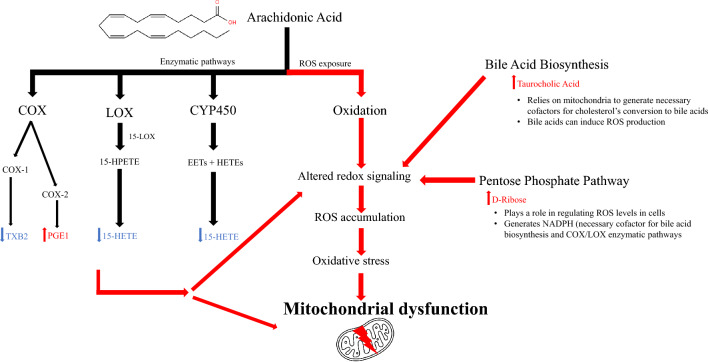
Proposed theoretical mechanism of perturbed pathways and mitochondrial dysfunction in disease syndromes of the black rhinoceros. Cycloxygenase (COX), lipoxygenase (LOX), and Cytochrome P450 (CYP450) represent enzymatic metabolism of arachidonic acid.

Arachidonic acid can be metabolized by multiple enzymatic pathways including the cyclooxygenases (COX), lipoxygenases (LOX), and cytochrome P450 (CYP450)^54^ (Fig. 5). In rodent models, unconjugated bile acids stimulate COX-2 expression^55^. In the present study, we detected both elevated levels of bile acids and the COX-2 metabolite PGE1. Simultaneously, we also observed decreased levels of TXB2, a COX-1 metabolite and 15-HETE a product of LOX and CYP450 metabolism. TXB2 is a pro-inflammatory eicosanoid with known roles in increasing platelet aggregation. Likewise, oxylipids such as 15-HETE are signaling molecules involved in regulation of inflammation^44^. Hence, down regulation of TXB2 and 15-HETE in black rhinos could suggest a compromise in their ability to respond to infections or inflammation. In addition to enzymatic pathways, arachidonic acid can be oxidized in the presence of reactive oxygen species (ROS) leading to suppression of the COX-1 pathway^43^. It is plausible that the perturbations observed in the arachidonic acid pathway are linked to both the enzymatic and oxidative metabolism of arachidonic acid.

Bile acids are synthesized in the liver from cholesterol. In addition to their role in nutrient absorption, both primary (chenodeoxycholic acid, cholic acid) and secondary bile acids (deoxycholic, and lithocholic acid) also function as signaling molecules that regulate glucose, lipid, and energy metabolism and participate in immunomodulation^56^. Bile acids can activate receptors that regulate leukocyte trafficking and macrophage differentiation, and thus modulate innate immunity^57^. In murine models, bile acids have been implicated in mitochondrial ROS production in hepatocytes^58^. The pentose phosphate pathway (PPP) plays a role in regulating cellular ROS levels. The PPP provides pentose sugars for ribonucleotide synthesis and generates nicotinamide adenine dinucleotide phosphate (NADPH) for reduction of glutathione and ROS^59^. NADPH is then used for fatty acid synthesis and scavenging of ROS^60^. NADPH is also a known cofactor for the conversion of cholesterol to bile acids^61^. These pathways appear implicated in altered reduction–oxidation (redox) homeostasis. Differential expression of taurocholic acid also is implicated in perturbations of the bile acid biosynthesis pathway. Several bile acids including glycocholic acid, deoxycholic acid, and cholic acid were up regulated in the inflammatory group. Previous research has shown that in the distal ileum and colon, bile acids regulate leukocyte trafficking and macrophage function through the activation of G-protein bile acid receptor 1 (GPBAR1) and Farnesoid-X-Receptor (FXR) which transforms this proinflammatory pathway into an anti-inflammatory response^57^. Taurocholic acid has previously been implicated as a promoting factor and biomarker of progression in liver cirrhosis; via the activation of hepatic stellate cells and up-regulation of TLR4 expression in humans^62^. However, although these metabolites met the fold-change criterion, they were not statistically significant possibly due to the relatively small sample size. Additional studies are warranted to compare these metabolites in a larger cohort of wild vs. ex-situ managed black rhinos.

D-ribose also has been implicated in perturbations in the pentose phosphate pathway. Deoxyribose 1-phosphate another compound involved in the pentose phosphate pathway was up-regulated (> 1.5) in the inflammatory group though this compound did not meet the statistical significance cut off (< 0.05). The pentose phosphate pathway is an important part of glucose metabolism as a major regulator for cellular redox homeostasis and biosynthesis^63^. This pathway contributes to the regulation of carbon homeostasis to provide nucleotide and amino acid precursors that provide reducing molecules for anabolism and to minimize oxidative stress^64^. The metabolic rerouting in oxidative and non-oxidative pentose phosphate pathway has key physiological roles in stabilizing the redox balance that facilitates the clearance of ROS^65^. The perturbations in arachidonic acid metabolism, bile acid biosynthesis, and the pentose phosphate pathway suggest that animals exhibiting inflammatory phenotypes may be susceptible to accumulation of ROS, oxidative stress, and ultimately mitochondrial dysfunction.

During nutrient excess, adipocytes can increase mitochondrial fatty acid oxidation^66^. This process eventually results in an increased electron supply to the electron transport chain of the mitochondria, the generation of ROS and ultimately oxidative stress^66^. The accumulation of ROS from perturbations to oxidative metabolism may be generating free radicals that damage proteins, lipids, and nucleic acids involved in cellular homeostasis. In humans, damage to mitochondrial DNA can result in the dysfunction of the mitochondrial electron transport chain (ETC), which creates a vicious cycle over time of exponentially increasing damage and dysfunction^67^. The pathways identified in our study suggest that perturbations are occurring within the metabolic networks that modulate mitochondrial function. The results of our study implicate mitochondrial and immune dysfunction in disease syndromes reported among ex-situ managed black rhinoceros.

## Limitations

There were six primary limitations in the present study. First, the health status comparison was inherently difficult to quantify. As it is unknown if the unusual disease syndromes observed ex-situ are different syndromes or rather varying manifestations of the same syndrome^2^. Second, we experienced gaps in our sample collection due to logistical challenges with research during the global pandemic. However, we had four samples for more than half and three samples for almost all the rhinos enrolled in this study, indicating that we have represented most of the longitudinal variation present during this study. Third, while the sex distribution was approximately even within our study, we observed disproportionately more males than females in the inflammatory phenotype cohort (Appendix S8). To date, only 22 SBR (11 males and 11 females) and 58 EBR (30 males and 28 females) exist ex-situ in the US and Mexico^6^. Of these animals, 1 SBR (female) and 1 EBR (male) are housed in Mexico^6^. Fourth, differences in husbandry practices can impact stress levels and subsequently risk of developing disease syndromes^35–38^. Moreover, alterations in adrenal activity have been previously associated with inflammatory disease phenotypes in black rhinos^40^. Fifth, omics studies are inherently exploratory and correlative. Further studies are required to validate and calibrate candidate biomarkers before they can be utilized in diagnostic tests. Additional -omics data also could provide a more enhanced physiological picture of the pathological perturbations underpinning the disease syndromes *ex-situ*. Sixth and finally, we do not have a true control population for this study. Comparison of wild versus ex-situ managed rhinos could facilitate improved characterization of correlations between metabolites to health status.

## Future directions

Future research on the molecular basis of inflammatory disease in black rhinos should consider the inclusion of samples from wild black rhinos to serve as controls. This information would be invaluable in deciphering which pathways are truly perturbed in ex-situ animals in the inflammatory phenotype cohort. Further research is also warranted to evaluate and validate the proposed candidate biomarkers mentioned above (Fig. 4). Analyses of the relationships among ROS, mitochondrial, and immune dysfunction could also generate new knowledge on physiological regulation in black rhinos. Like black rhinos, humans also develop chronic liver diseases. In humans, this process is characterized by a proinflammatory cascade that is activated in the liver and stimulates the circulation of innate immune cells^68^. Therefore, future research should characterize rhinoceros immune cells and their possible roles in immunomodulation and pathogenesis. Interestingly, previous research suggests that arachidonic acid and the presence of excessive iron may induce mitochondrial dysfunction^51^. This phenomenon should be explored in black rhinos, as the ex-situ population has a long history of developing iron overload disease (IOD)^3,69–72^. Future comparisons should be made between metabolomic profiles of multiple rhinoceros species including IOD-susceptible black rhinoceros, and IOD-resistant species white rhinoceros (*Ceratotherium simum*) and greater one-horned (GOH) rhinoceros (*Rhinoceros unicornis*)^33^. Lastly, a multiomic database that robustly characterizes molecular networks and resulting disease phenotypes in this (and other) species should be developed. This database should facilitate a more thorough exploration of black rhino disease syndrome etiologies.

## Conclusions

As the first characterization of the black rhinoceros metabolome, this research provides foundational baseline information on their serum metabolites by subspecies, sex, and health phenotype. This information will be incorporated into a digital biobank at the Smithsonian National Zoo & Conservation Biology Institute so that future research can be computationally conducted to enhance our understanding of rhinoceros health. With our new ability to begin to digitize disease-related data, we hope this study will provide the baseline information required for future biomarker discovery, validation, and calibration so that new diagnostic and treatment options will become available to enhance the health and welfare of black rhinos managed ex-situ.

## Methods

### Animals and sample collection

Our study population comprised of fourteen EBR (*D. b. michaeli*) and sixteen SBR (*Diceros b. minor*) housed in 15 zoological institutions or private ranches in the United States. Ninety-seven serum samples were collected in a prospective longitudinal study (approximately every 3 months). Animals ranged in age from 3 to 33 years old, and the median age was 21. When possible, four longitudinal samples were collected throughout one year. However, this was not always possible due to logistical challenges associated with conducting research during a global pandemic and with gathering blood samples prospectively via voluntary blood draws. This project was reviewed and approved by the Smithsonian National Zoo and Conservation Biology Institute’s Animal Care and Use Committee; approval number ACUC # 19-28). Serum samples were collected by the veterinary staff at participating institutions and shipped to the Smithsonian’s National Zoo & Conservation Biology Institute (Front Royal, VA) overnight on ice. Upon receipt, samples were aliquoted (0.5–2.0 mL) and stored at − 80 °C until analysis.

### Survey

We surveyed black rhinoceros-housing institutions using the Qualtrics survey software. Information collected included data pertaining to the health and reproductive history, nutrition, and husbandry of each rhino enrolled in this study. A sample survey is included in supplementary data (Appendix S2).

### Sample preparation

The serum aliquots (0.1 mL) were shipped to an accredited commercial laboratory (Creative Proteomics, Shirley, NY, USA) for untargeted metabolomic analysis. Samples were processed using the external laboratory’s standard techniques for LC–MS analysis (see supplemental materials S7 for details). Quality control samples were included in the run. Three analytical comparisons were made: (1) subspecies, (2) sex, and (3) health status.

### Instruments and materials

Samples were analyzed using an Ultimate 3000LC combined with the ThermoQ Exactive MS platform, Temp Functional Centrifugation (Eppendorf), ACQUITY UPLC HSS T3 (100 × 2.1 mm × 1.8 µm) and reagents (all from Merck) included acetonitrile, methanol, formic acid, and DL-o-chlorophenylalanine.

### UPLC-MS

Separation was performed by Ultimate 3000LC combined with ThermoQ Exactive MS which uses accurate MS1 profiling followed by MS2 for feature peak identification and then screened with ESI–MS. The LC system comprised of an ACQUITY UPLC HSS T3 (100 × 2.1 mm × 1.8 µm) with Ultimate 3000LC. The mobile phase composed of solvent A (0.05% formic acid in water) and solvent B (acetonitrile) with a gradient elution (0–1 min, 95%A, 1–12 min, 95%–5% A, 12–13.5 min, 5% A, 13.5–13.6 min, 5–95% A, 13.6–16 min, 95% A) (Appendix S7). The flow rate of the mobile phase was 0.3 mL per min. The column temperature was maintained at 40 °C and sample manager temperature was set at 4 °C. Global metabolomic profiling requires both positive and negative ion mode.

### Statistical analysis

Compound Discover (3.0, Thermo) was used for acquiring and aligning metabolites as well as metabolite identification from raw data based on retention time of the ion signals, MS1, and MS/MS. The SIMCA-P program (v. 14.1) was used to merge and import ESI− and ESI+ ions for data visualization and outlier identification. Supervised regression modeling was performed using partial least squares discriminant analysis (PLS-DA) or orthogonal partial least squares discriminant analysis (OPLS-DA) to identify potential biomarkers. The biomarkers were filtered and confirmed by combining the results of the VIP values (VIP > 1.5) and t-test (*p* < 0.05). Metabolites were included in analysis if expressed in all serum samples, 636 metabolites were expressed in all samples (352 ESI− and 284 ESI+). Corresponding spectral binning values were normalized for subsequent analyses. Each of the covariate comparisons determined the extent of differential expression by first calculating the fold change within each cohort comparison, second taking the fold change, and then calculating two-sided t-tests assuming unequal variance. Hierarchical cluster analysis was then performed to generate a list of metabolites for each covariate comparison. The resulting metabolites were included in the differential expression tables. We used the MetaboAnalyst 5.0 software^34^ to generate (1) ordination plots including PCA and OPLS-DA, (2) VIP scores plots, (3) volcano plots of differentially expressed compounds, (4) run quantitative enrichment analyses for affected metabolite and lipid pathways, and (5) run biomarker analyses using a receiver operating characteristic (ROC) curve-based approach for identifying candidate biomarkers using area under the curve (AOC).

### Supplementary Information

Supplementary Information.

## Data Availability

Data is currently maintained through the Smithsonian’s National Zoo & Conservation Biology Institute, Center for Species Survival Digital Biobank. These are in the Black Rhino Health Project Metabolomics Repository and include the .RAW files associated with each serum sample, metadata, and .csv files generated from Compound Discover (Thermo, 3.0) and SIMCA-P program (v. 14.1) with proteins identified and corresponding spectral binning signals. Data can be made available upon request.
