# Supplementary material for: Metabolomic profiling implicates mitochondrial and immune dysfunction in disease syndromes of the critically endangered black rhinoceros (Diceros bicornis)
Source: Sci Rep. 2023 Sep 19;13:15464. doi: 10.1038/s41598-023-41508-4 (PMC10509206; doi:10.1038/s41598-023-41508-4)
Supplement: Supplementary file 1 — Supplementary Information. [file 41598_2023_41508_MOESM1_ESM.pdf]

Supplemental Materials

Metabolomic profiling implicates mitochondrial and immune dysfunction in disease syndromes of the critically endangered black rhinoceros (*Diceros bicornis*)

Molly L. Corder[1,2], Emanuel Petricoin[3], Yue Li[4], Timothy Cleland[5], Alexandra DeCandia[6,7], A. Alonso Aguirre[8], Budhan S. Pukazhenthi[1,*]

[1]Smithsonian's National Zoo and Conservation Biology Institute, Center for Species Survival, Front Royal, 22630, USA

[2] George Mason University, Department of Environmental Science & Policy, Fairfax, 22030, USA

[3]George Mason University, Center for Applied Proteomics and Molecular Medicine, Manassas, 20900, USA

[4]University of Maryland, Department of Chemistry and Biochemistry, College Park, MD 20742, USA

[5]Smithsonian Museum Conservation Institute, Suitland, MD, 20746, USA

[6]Georgetown University, Department of Biology, Washington, DC, 20057, USA

[7]Smithsonian's National Zoo and Conservation Biology Institute, Center for Conservation Genomics, Washington, DC, 20008, USA

[8]Colorado State University, Warner College of Natural Resources, Fort Collins, 80523, USA

*Corresponding author:
Budhan Pukazhenthi
Smithsonian's National Zoo & Conservation Biology Institute
1500 Remount Road, Front Royal, VA 22630
PukazhenthiB@si.edu

n	*Disease Phenotypes*
9	Dental/periodontal
7	Chronic inflammation
7	Lameness
6	Suspected iron overload disease (IOD)*
6	Chronic intermittent nose bleeds
4	Ulcerative dermatitis
3	Metabolic disease
3	Reproductive dysfunction
2	Anemia
2	Renal disease
2	Chronic intermittent diarrhea
2	Immune dysfunction
1	Hepatic disease
1	Idiopathic hemorrhagic vasculopathy syndrome (IHVS)
1	Pododermatitis

Table S1. Disease phenotypes reported by housing institutions (2019-2021) where *n*=number of animals out of 30 individuals with history of reported disease phenotype. Three animals were omitted from the study if they did not have clinical signs of inflammation (i.e., only reproductive dysfunction reported). *IOD cannot be reliably diagnosed pre-mortem.

Survey S2 Blank Qualtrics Survey

Health Survey: general health, reproductive status, diet, and husbandry

Introduction

Q1. Thank you for your dedication to black rhinoceros conservation and your willingness to work with us on this project. Throughout this black rhinoceros health study, we ask all collaborators to complete an enrollment survey followed by 4 quarterly follow-up surveys. This qualitative data will be used to interpret the health data generated. Quarterly surveys should be submitted on either the day before or day of quarterly sample collection.

This enrollment survey consists of four sections:

1) General Health
2) Reproductive Status
3) Diet
4) Husbandry

General _Health_Parameters

Q2. **Section 1: General Health**

Q3. What is the animal's name and studbook number?

[ ]

Q4. What is the animal's sex?

Male

Female

Q5. Date of birth:

Q6. What is the current, last recorded, or estimated weight (in pounds)?

Q7. Height (*measured to the withers in inches/feet*)

Q8. Does this rhino have/have a history of any of the following health conditions? *If yes, please describe.*

	No	Yes, current condition	Yes, within the last 4+ weeks	Yes, within the last year	Yes, over 1 year ago
Obesity	○	○	○	○	○
Insulin resistance	○	○	○	○	○
Chronic inflammation	○	○	○	○	○
Ulcerative dermatitis	○	○	○	○	○
Metabolic disease	○	○	○	○	○
Immune dysfunction	○	○	○	○	○

	No	Yes, current condition	Yes, within the last 4+ weeks	Yes, within the last year	Yes, over 1 year ago
Iron overload disease	○	○	○	○	○
Lameness	○	○	○	○	○
Reproductive dysfunction	○	○	○	○	○
Hepatic disease	○	○	○	○	○
Dental/periodontal disease	○	○	○	○	○
Anemia	○	○	○	○	○
Renal disease	○	○	○	○	○
Intermittent chronic diarrhea	○	○	○	○	○
Intermittent nose bleeds	○	○	○	○	○
Intermittent tail bleeding	○	○	○	○	○
Other (please describe)	○	○	○	○	○

Q9. Is this animal currently being treated with antibiotics?

Yes (please list medication, dosage, and reason for administration)

No

Q10. Please describe any current medications or treatments (*Please specify what each is for*):

Medications with specific dosages

Supplements with specific dosages

Other treatments

Q11. Any additional health comments?

Reproductive_Status

Q12. **Section 2: Reproductive Status**

Q13. Does this animal have any current breeding recommendations?

Yes

No

Q14. Has this animal been introduced to another animal for breeding?

No

Yes, in the last month (please specify date)

Yes, in the last year (please specify date)

Q15. Has mating been observed?

Yes (please describe)

No

Q16. Is the animal currently enrolled in any endocrine or ultrasound studies?

Yes, both endocrine and ultrasound studies (please describe studies and participants)

Endocrine studies only (please describe studies and participants)

Ultrasound studies only (please describe studies and participants)

No, not enrolled in any endocrine or ultrasound studies

Q17. Has this animal had any assisted reproductive procedures in the last 4 weeks (such as artificial insemination, semen collection, etc)?

Yes (please describe)

No

Q18. FEMALES - Does this animal have any history of producing offspring?

No, never bred.

Yes, pregnancy occurred but was not carried to term.

Yes, pregnancy and parturition occurred, but calf died soon after.

Yes, healthy calf/calves produced (If multiple pregnancies, please describe outcomes)

N/A animal is male

Q19. MALES - Does this animal have any history of producing offspring?

No, no attempts to breed naturally or collect semen

No, natural breeding attempted but not successful (please describe)

No, semen collected, but no resulting pregnancies

Yes, an offspring was produced after natural breeding

Yes, an offspring was produced after assisted reproduction (please describe)

Q20. Any additional reproductive comments?

Q21. **Section 3: Diet**

Q22. Approximately how much of this rhino's diet consists of the following items on average?

Dietary Composition (%)

0 10 20 30 40 50 60 70 80 90 100

Browse (please describe types offered) 0

Hay (please describe types offered) 0

Pellets (please describe types offered) 0

Other (please describe types offered) 0

Q23. Please provide the following dietary samples from the diet fed to this rhino the day before blood and fecal sample collection: 100 grams of browse, 100 grams of hay, and 50 grams of grain. Please note the species/types of each dietary item provided. Samples should be frozen in plastic bags provided and stored in -80 C freezer until the end of the study.

Browse, 100 grams (please specify type)

Hay, 100 grams (please specify type)

Pellets, 50 grams (please specify type)

Please list any other diet items. Samples are not required for training treats.

Q24. Approximately what quantity of each food item is the animal offered each day? *Please include units (i.e. pounds of specific food items)*

Browse

Hay

Pellets

Other

Q25. Any additional dietary comments? Please indicate if the animal receives any dietary supplements (note supplement type, dosage, and frequency).

Husbandry_Data

Q26. **Section 4: Husbandry**

Q27. Does this animal have any known behavioral stereotypies or unusual behaviors? If yes, please describe.

Yes

Maybe

No

Q28. Do you consider this animal to be chronically stressed? Please describe

	Definitely yes	Probably yes	Might or might not	Probably not	Definitely not
This animal is chronically stressed.	O	O	O	O	O

	Definitely yes	Probably yes	Might or might not	Probably not	Definitely not
This animal is sometimes stressed.	O	O	O	O	O
This animal is never stressed.	O	O	O	O	O

Q29. This rhino spends approximately what percent of their daily time in the following places:

Outside on exhibit	0
Outside off exhibit	0
Inside on exhibit	0
Inside off exhibit	0
Total	0

Q30. This animal is housed alone.

Yes

Sometimes (please describe)

No (please describe)

Q31. This animal has visual access to other animals daily.

Yes (please describe)

Sometimes (please describe)

No

Q32. Please describe the animal's daily environment (include approximate enclosure sizes/objects present)

Q33. How much of the animal's time is spent in the following ways?

	None at all		A little		A moderate amount		A lot		A great deal		
	0	10	20	30	40	50	60	70	80	90	100
With other animals											
With enrichment items											
In training sessions											
On exhibit											
Off exhibit											

Q34. Please describe any current training routines or training plans in progress.

Q35. Any additional husbandry comments?

End of Survey

Q42. **Thank you for taking the time to complete the black rhinoceros healthy survey! Please click the "Submit Survey" button to submit your survey.**

All answers are saved automatically before you click the "Submit Survey" button. You can re-open the survey and come back to your saved responses as long as you do so **before** the submitting the survey. If you accidentally click the "Submit Survey" button before all answers are filled in, please contact Molly Corder at CorderM@si.edu to request a new survey link.

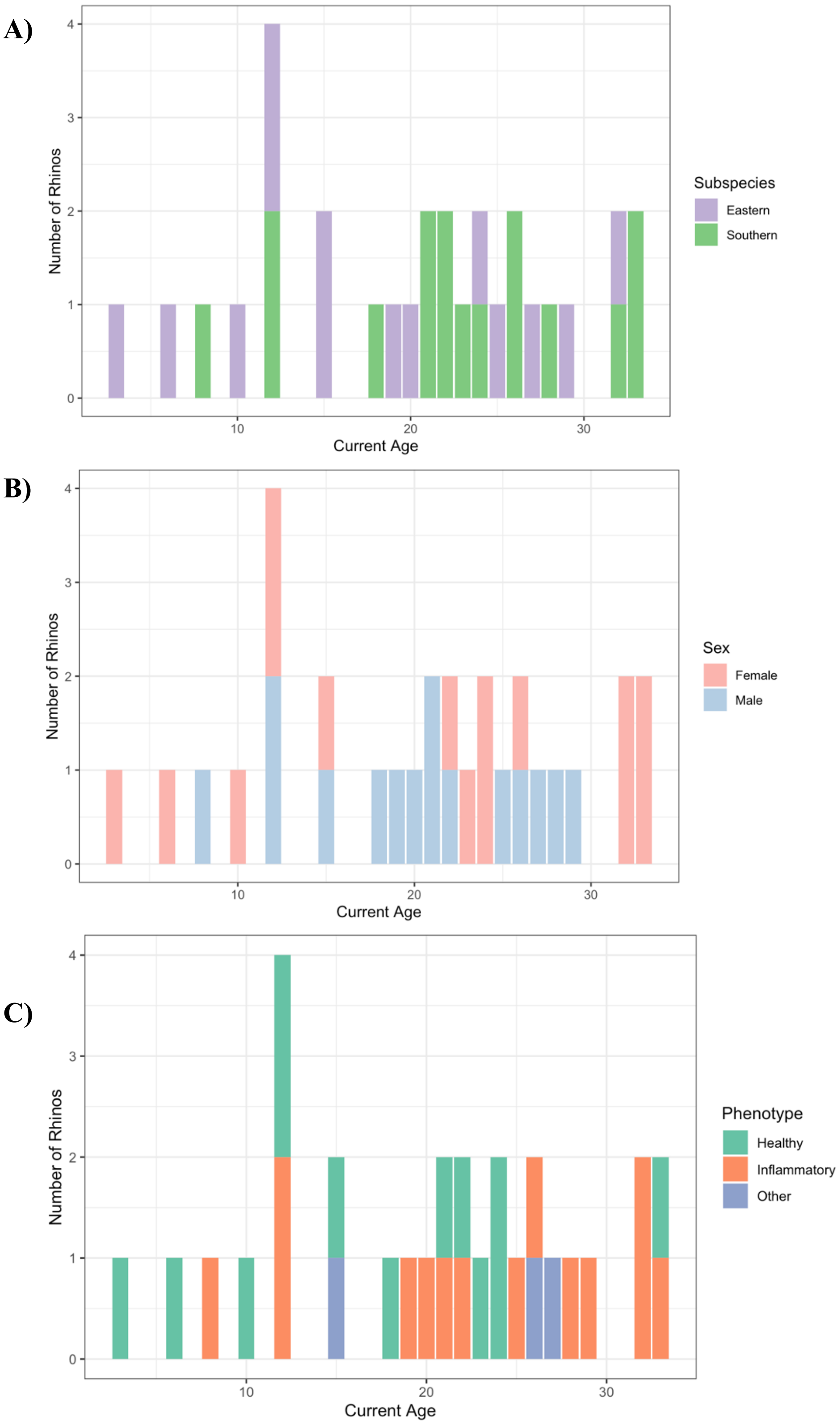

Figure S3 Bar plot Age distribution of black rhinoceros (n=30) housed *ex-situ* by population demographics: A) subspecies, B) sex, and C) health status.

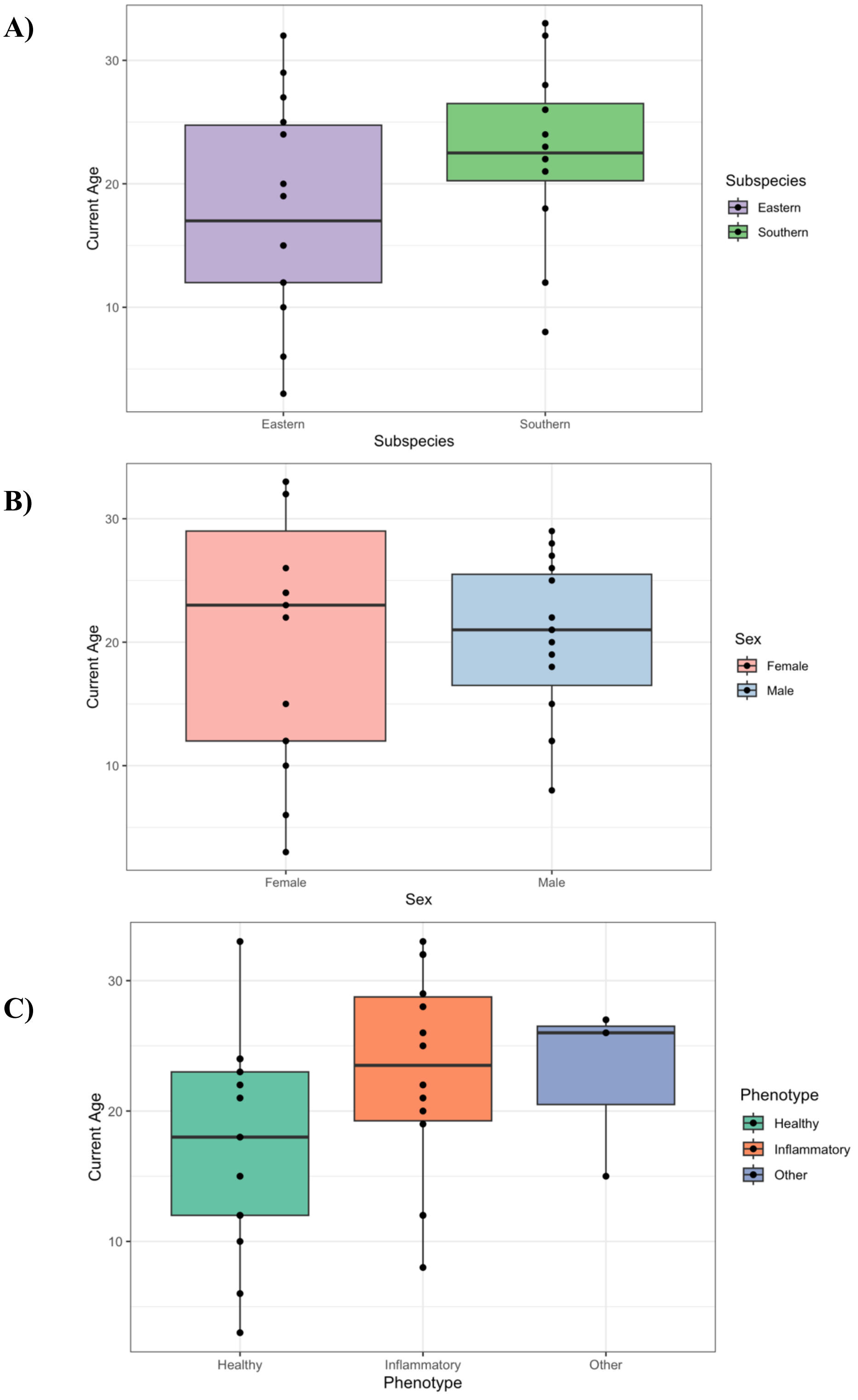

Figure S4 Box plot age distribution of black rhinoceros (n=30) housed *ex-situ* by population demographics: A) subspecies, B) sex, and C) health status.

A)

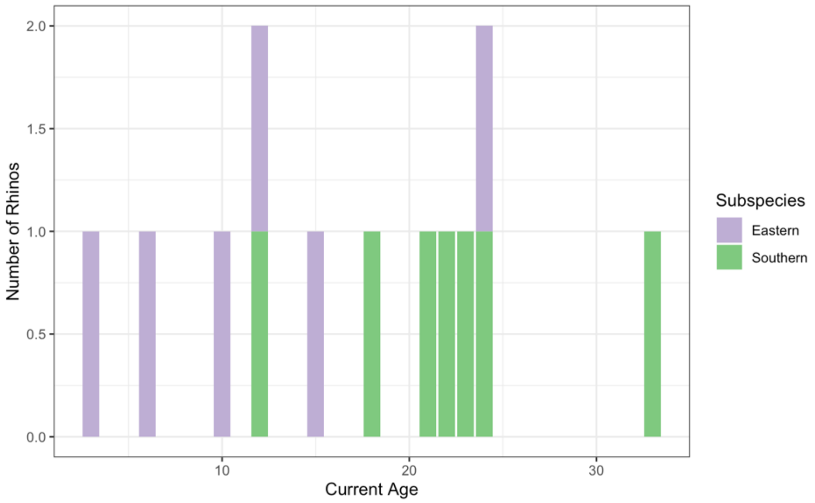

B)

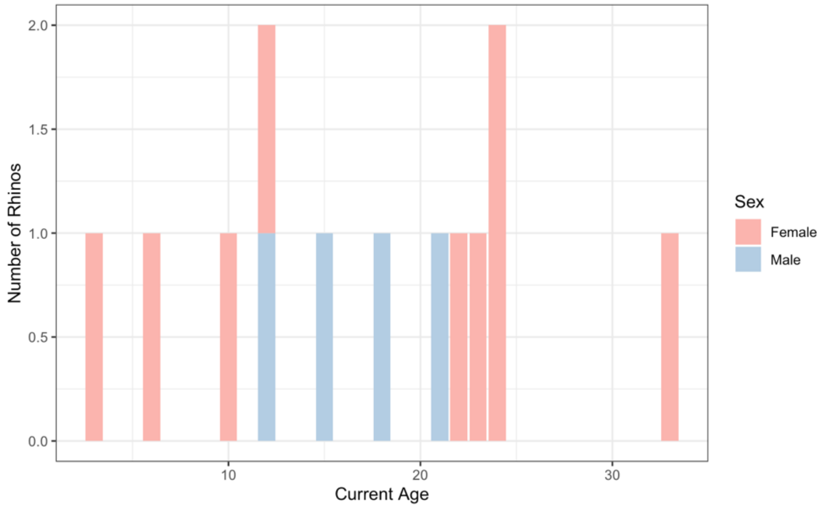

C)

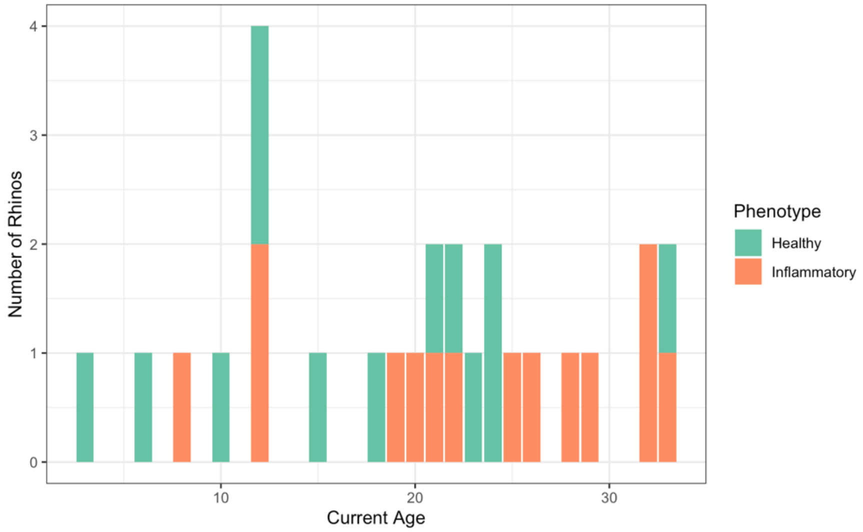

Figure S5 Bar plots of age distribution of black rhinoceros (n=27; healthy=13, inflamed=14) *ex-situ* by population demographics: A) subspecies, B) sex, and C) health status.

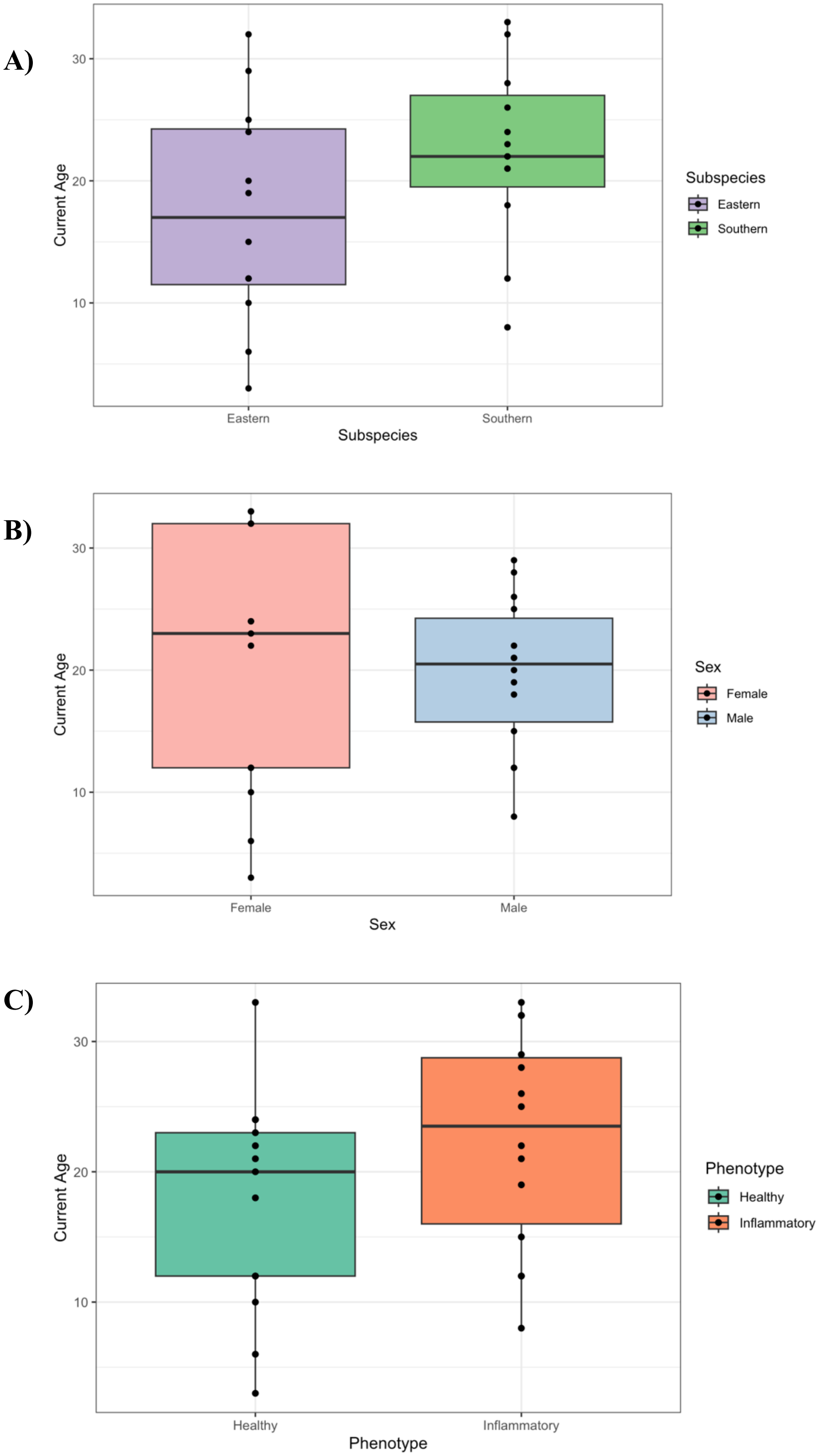

Figure S6 Boxplots of age distribution of black rhinoceros (n=27; healthy=13, inflamed=14) *ex-situ* by population demographics: A) subspecies, B) sex, and C) health status.

RT(min)	A%	B%
0	95	5
1	95	5
12	5	95
13.5	5	95
13.6	95	5
16	95	5

Table S7: *UPLC-MS:* The mobile phase for the LC method is composed of solvent A (0.05% formic acid water) and solvent B (acetonitrile) with a gradient elution (0-1 min, 95%A, 1-12 min, 95%-5% A, 12-13.5 min, 5% A,13.5-13.6 min,5%-95% A, 13.6-16 min, 95% A). The flow rate of the mobile phase was 0.3 mL per min.

	Eastern Subspecies (EBR)		Southern Subspecies (SBR)	
	Male	Female	Male	Female
Healthy	1	5	3	4
Inflamed	5	1	5	3

Table S8: Metadata for 27 animals included in healthy vs. inflamed metabolomic comparison.
